# Supplementary material for: Substrate Type and Concentration Differently Affect Colon Cancer Cells Ultrastructural Morphology, EMT Markers, and Matrix Degrading Enzymes
Source: Biomolecules. 2022 Nov 30;12(12):1786. doi: 10.3390/biom12121786 (PMC9775446; doi:10.3390/biom12121786)
Supplement: Supplementary file 1 [file biomolecules-12-01786-s001.zip › biomolecules-2003801-supplementary.pdf]

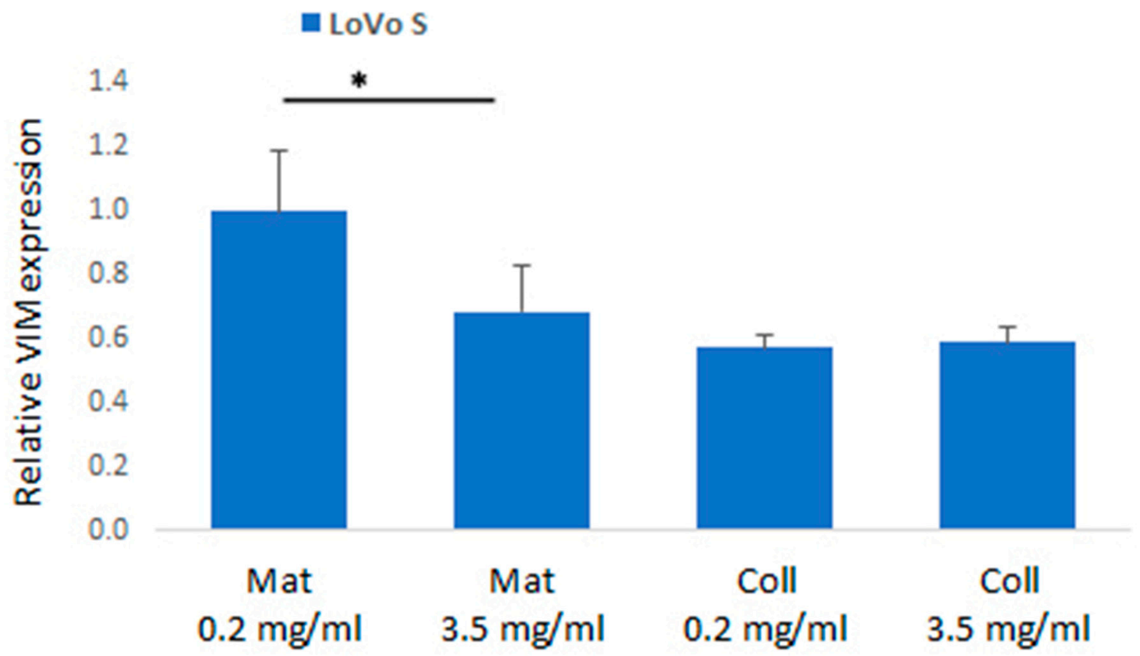

Figure S1. Evaluation of VIM expression in LoVo-S cells. VIM expression decreases from standard Matrigel (0.2 mg/mL) to both different concentrations of collagen. * $p \leq 0.05$.
